# Supplementary material for: Pyruvate Dehydrogenase Kinase 1 inhibition mediated oxidative phosphorylation enhancement in cartilage promotes osteoarthritis progression
Source: BMC Musculoskelet Disord. 2023 Jul 20;24:597. doi: 10.1186/s12891-023-06585-6 (PMC10357736; doi:10.1186/s12891-023-06585-6)
Supplement: Supplementary file 1 — Additional file 1. [file 12891_2023_6585_MOESM1_ESM.zip › KEGG.Gsea.1653622432464/KEGG_WNT_SIGNALING_PATHWAY.html]

Details for gene set KEGG\_WNT\_SIGNALING\_PATHWAY[GSEA]

|  || Dataset | 55457\_collapsed\_to\_symbols.cls.cls #OA\_versus\_normal.cls.cls #OA\_versus\_normal\_repos |
| Phenotype | cls.cls#OA\_versus\_normal\_repos |
| Upregulated in class | normal |
| GeneSet | KEGG\_WNT\_SIGNALING\_PATHWAY |
| Enrichment Score (ES) | -0.2692958 |
| Normalized Enrichment Score (NES) | -1.2275203 |
| Nominal p-value | 0.105421685 |
| FDR q-value | 0.3358861 |
| FWER p-Value | 1.0 |
Table: GSEA Results Summary

  

Fig 1: Enrichment plot: KEGG\_WNT\_SIGNALING\_PATHWAY      
 Profile of the Running ES Score & Positions of GeneSet Members on the Rank Ordered List

  

| SYMBOL | TITLE | RANK IN GENE LIST | RANK METRIC SCORE | RUNNING ES | CORE ENRICHMENT || 1 | DKK2 | dickkopf WNT signaling pathway inhibitor 2 [Source:HGNC Symbol;Acc:HGNC:2892] | 33 | 1.033 | 0.0204 | No |
| 2 | FZD1 | frizzled class receptor 1 [Source:HGNC Symbol;Acc:HGNC:4038] | 111 | 0.898 | 0.0344 | No |
| 3 | WIF1 | WNT inhibitory factor 1 [Source:HGNC Symbol;Acc:HGNC:18081] | 231 | 0.799 | 0.0429 | No |
| 4 | FZD7 | frizzled class receptor 7 [Source:HGNC Symbol;Acc:HGNC:4045] | 274 | 0.768 | 0.0568 | No |
| 5 | SMAD2 | SMAD family member 2 [Source:HGNC Symbol;Acc:HGNC:6768] | 275 | 0.768 | 0.0739 | No |
| 6 | WNT5B | Wnt family member 5B [Source:HGNC Symbol;Acc:HGNC:16265] | 327 | 0.734 | 0.0863 | No |
| 7 | RBX1 | ring-box 1 [Source:HGNC Symbol;Acc:HGNC:9928] | 348 | 0.724 | 0.1008 | No |
| 8 | PSEN1 | presenilin 1 [Source:HGNC Symbol;Acc:HGNC:9508] | 425 | 0.695 | 0.1104 | No |
| 9 | CACYBP | calcyclin binding protein [Source:HGNC Symbol;Acc:HGNC:30423] | 447 | 0.683 | 0.1240 | No |
| 10 | MAPK9 | mitogen-activated protein kinase 9 [Source:HGNC Symbol;Acc:HGNC:6886] | 721 | 0.588 | 0.1157 | No |
| 11 | PRKACB | protein kinase cAMP-activated catalytic subunit beta [Source:HGNC Symbol;Acc:HGNC:9381] | 771 | 0.576 | 0.1247 | No |
| 12 | CAMK2A | calcium/calmodulin dependent protein kinase II alpha [Source:HGNC Symbol;Acc:HGNC:1460] | 916 | 0.538 | 0.1254 | No |
| 13 | TBL1XR1 | TBL1X receptor 1 [Source:HGNC Symbol;Acc:HGNC:29529] | 1070 | 0.501 | 0.1246 | No |
| 14 | FZD2 | frizzled class receptor 2 [Source:HGNC Symbol;Acc:HGNC:4040] | 1111 | 0.494 | 0.1325 | No |
| 15 | MAP3K7 | mitogen-activated protein kinase kinase kinase 7 [Source:HGNC Symbol;Acc:HGNC:6859] | 1701 | 0.399 | 0.0952 | No |
| 16 | CSNK2B | casein kinase 2 beta [Source:HGNC Symbol;Acc:HGNC:2460] | 1788 | 0.385 | 0.0971 | No |
| 17 | WNT5A | Wnt family member 5A [Source:HGNC Symbol;Acc:HGNC:12784] | 1995 | 0.361 | 0.0890 | No |
| 18 | WNT3 | Wnt family member 3 [Source:HGNC Symbol;Acc:HGNC:12782] | 2003 | 0.360 | 0.0965 | No |
| 19 | PPP2R5D | protein phosphatase 2 regulatory subunit B'delta [Source:HGNC Symbol;Acc:HGNC:9312] | 2137 | 0.346 | 0.0938 | No |
| 20 | PLCB4 | phospholipase C beta 4 [Source:HGNC Symbol;Acc:HGNC:9059] | 2217 | 0.337 | 0.0951 | No |
| 21 | SIAH1 | siah E3 ubiquitin protein ligase 1 [Source:HGNC Symbol;Acc:HGNC:10857] | 2389 | 0.315 | 0.0887 | No |
| 22 | DAAM1 | dishevelled associated activator of morphogenesis 1 [Source:HGNC Symbol;Acc:HGNC:18142] | 2402 | 0.314 | 0.0948 | No |
| 23 | SFRP1 | secreted frizzled related protein 1 [Source:HGNC Symbol;Acc:HGNC:10776] | 2503 | 0.303 | 0.0937 | No |
| 24 | CCND2 | cyclin D2 [Source:HGNC Symbol;Acc:HGNC:1583] | 2508 | 0.302 | 0.1001 | No |
| 25 | VANGL1 | VANGL planar cell polarity protein 1 [Source:HGNC Symbol;Acc:HGNC:15512] | 2541 | 0.298 | 0.1043 | No |
| 26 | PPP2R5A | protein phosphatase 2 regulatory subunit B'alpha [Source:HGNC Symbol;Acc:HGNC:9309] | 2592 | 0.291 | 0.1068 | No |
| 27 | DVL2 | dishevelled segment polarity protein 2 [Source:HGNC Symbol;Acc:HGNC:3086] | 2693 | 0.280 | 0.1053 | No |
| 28 | WNT2 | Wnt family member 2 [Source:HGNC Symbol;Acc:HGNC:12780] | 2700 | 0.279 | 0.1110 | No |
| 29 | CAMK2G | calcium/calmodulin dependent protein kinase II gamma [Source:HGNC Symbol;Acc:HGNC:1463] | 2900 | 0.261 | 0.1012 | No |
| 30 | NFATC1 | nuclear factor of activated T cells 1 [Source:HGNC Symbol;Acc:HGNC:7775] | 3027 | 0.247 | 0.0969 | No |
| 31 | SENP2 | SUMO specific peptidase 2 [Source:HGNC Symbol;Acc:HGNC:23116] | 3040 | 0.246 | 0.1014 | No |
| 32 | NFATC3 | nuclear factor of activated T cells 3 [Source:HGNC Symbol;Acc:HGNC:7777] | 3079 | 0.242 | 0.1038 | No |
| 33 | PPP2R5C | protein phosphatase 2 regulatory subunit B'gamma [Source:HGNC Symbol;Acc:HGNC:9311] | 3205 | 0.230 | 0.0992 | No |
| 34 | PLCB1 | phospholipase C beta 1 [Source:HGNC Symbol;Acc:HGNC:15917] | 3724 | 0.183 | 0.0627 | No |
| 35 | PRKCA | protein kinase C alpha [Source:HGNC Symbol;Acc:HGNC:9393] | 3728 | 0.183 | 0.0665 | No |
| 36 | PPP3CA | protein phosphatase 3 catalytic subunit alpha [Source:HGNC Symbol;Acc:HGNC:9314] | 3803 | 0.176 | 0.0647 | No |
| 37 | RAC3 | Rac family small GTPase 3 [Source:HGNC Symbol;Acc:HGNC:9803] | 3849 | 0.171 | 0.0649 | No |
| 38 | SMAD3 | SMAD family member 3 [Source:HGNC Symbol;Acc:HGNC:6769] | 4061 | 0.150 | 0.0518 | No |
| 39 | PPP2CA | protein phosphatase 2 catalytic subunit alpha [Source:HGNC Symbol;Acc:HGNC:9299] | 4146 | 0.143 | 0.0484 | No |
| 40 | WNT2B | Wnt family member 2B [Source:HGNC Symbol;Acc:HGNC:12781] | 4194 | 0.138 | 0.0478 | No |
| 41 | LRP5 | LDL receptor related protein 5 [Source:HGNC Symbol;Acc:HGNC:6697] | 4305 | 0.128 | 0.0420 | No |
| 42 | CCND3 | cyclin D3 [Source:HGNC Symbol;Acc:HGNC:1585] | 4426 | 0.119 | 0.0352 | No |
| 43 | DVL1 | dishevelled segment polarity protein 1 [Source:HGNC Symbol;Acc:HGNC:3084] | 4612 | 0.104 | 0.0231 | No |
| 44 | WNT11 | Wnt family member 11 [Source:HGNC Symbol;Acc:HGNC:12776] | 4688 | 0.097 | 0.0194 | No |
| 45 | TCF7L2 | transcription factor 7 like 2 [Source:HGNC Symbol;Acc:HGNC:11641] | 5034 | 0.070 | -0.0061 | No |
| 46 | CTBP2 | C-terminal binding protein 2 [Source:HGNC Symbol;Acc:HGNC:2495] | 5066 | 0.067 | -0.0070 | No |
| 47 | PPP3CB | protein phosphatase 3 catalytic subunit beta [Source:HGNC Symbol;Acc:HGNC:9315] | 5311 | 0.046 | -0.0251 | No |
| 48 | FZD5 | frizzled class receptor 5 [Source:HGNC Symbol;Acc:HGNC:4043] | 5392 | 0.041 | -0.0305 | No |
| 49 | SFRP4 | secreted frizzled related protein 4 [Source:HGNC Symbol;Acc:HGNC:10778] | 5443 | 0.037 | -0.0335 | No |
| 50 | TP53 | tumor protein p53 [Source:HGNC Symbol;Acc:HGNC:11998] | 5511 | 0.032 | -0.0381 | No |
| 51 | CTNNBIP1 | catenin beta interacting protein 1 [Source:HGNC Symbol;Acc:HGNC:16913] | 5731 | 0.013 | -0.0549 | No |
| 52 | WNT6 | Wnt family member 6 [Source:HGNC Symbol;Acc:HGNC:12785] | 5781 | 0.010 | -0.0586 | No |
| 53 | FRAT1 | FRAT regulator of WNT signaling pathway 1 [Source:HGNC Symbol;Acc:HGNC:3944] | 6027 | -0.007 | -0.0776 | No |
| 54 | FZD8 | frizzled class receptor 8 [Source:HGNC Symbol;Acc:HGNC:4046] | 6084 | -0.011 | -0.0818 | No |
| 55 | RAC2 | Rac family small GTPase 2 [Source:HGNC Symbol;Acc:HGNC:9802] | 6098 | -0.013 | -0.0825 | No |
| 56 | CSNK2A2 | casein kinase 2 alpha 2 [Source:HGNC Symbol;Acc:HGNC:2459] | 6105 | -0.013 | -0.0827 | No |
| 57 | WNT7B | Wnt family member 7B [Source:HGNC Symbol;Acc:HGNC:12787] | 6230 | -0.023 | -0.0919 | No |
| 58 | DKK1 | dickkopf WNT signaling pathway inhibitor 1 [Source:HGNC Symbol;Acc:HGNC:2891] | 6392 | -0.034 | -0.1037 | No |
| 59 | PRKCB | protein kinase C beta [Source:HGNC Symbol;Acc:HGNC:9395] | 6453 | -0.039 | -0.1076 | No |
| 60 | FZD9 | frizzled class receptor 9 [Source:HGNC Symbol;Acc:HGNC:4047] | 6812 | -0.064 | -0.1342 | No |
| 61 | PPP3CC | protein phosphatase 3 catalytic subunit gamma [Source:HGNC Symbol;Acc:HGNC:9316] | 7031 | -0.082 | -0.1494 | No |
| 62 | PLCB2 | phospholipase C beta 2 [Source:HGNC Symbol;Acc:HGNC:9055] | 7052 | -0.084 | -0.1491 | No |
| 63 | PLCB3 | phospholipase C beta 3 [Source:HGNC Symbol;Acc:HGNC:9056] | 7118 | -0.090 | -0.1522 | No |
| 64 | WNT7A | Wnt family member 7A [Source:HGNC Symbol;Acc:HGNC:12786] | 7166 | -0.093 | -0.1538 | No |
| 65 | RAC1 | Rac family small GTPase 1 [Source:HGNC Symbol;Acc:HGNC:9801] | 7192 | -0.096 | -0.1537 | No |
| 66 | PPP2CB | protein phosphatase 2 catalytic subunit beta [Source:HGNC Symbol;Acc:HGNC:9300] | 7265 | -0.100 | -0.1571 | No |
| 67 | APC2 | APC regulator of WNT signaling pathway 2 [Source:HGNC Symbol;Acc:HGNC:24036] | 7271 | -0.101 | -0.1552 | No |
| 68 | NLK | nemo like kinase [Source:HGNC Symbol;Acc:HGNC:29858] | 7336 | -0.105 | -0.1579 | No |
| 69 | TCF7L1 | transcription factor 7 like 1 [Source:HGNC Symbol;Acc:HGNC:11640] | 7383 | -0.109 | -0.1591 | No |
| 70 | LEF1 | lymphoid enhancer binding factor 1 [Source:HGNC Symbol;Acc:HGNC:6551] | 7467 | -0.116 | -0.1630 | No |
| 71 | RUVBL1 | RuvB like AAA ATPase 1 [Source:HGNC Symbol;Acc:HGNC:10474] | 7477 | -0.116 | -0.1611 | No |
| 72 | CCND1 | cyclin D1 [Source:HGNC Symbol;Acc:HGNC:1582] | 7512 | -0.119 | -0.1611 | No |
| 73 | FRAT2 | FRAT regulator of WNT signaling pathway 2 [Source:HGNC Symbol;Acc:HGNC:16048] | 7630 | -0.129 | -0.1674 | No |
| 74 | WNT10B | Wnt family member 10B [Source:HGNC Symbol;Acc:HGNC:12775] | 7700 | -0.134 | -0.1698 | No |
| 75 | DKK4 | dickkopf WNT signaling pathway inhibitor 4 [Source:HGNC Symbol;Acc:HGNC:2894] | 8048 | -0.159 | -0.1934 | No |
| 76 | FZD4 | frizzled class receptor 4 [Source:HGNC Symbol;Acc:HGNC:4042] | 8159 | -0.168 | -0.1983 | No |
| 77 | DAAM2 | dishevelled associated activator of morphogenesis 2 [Source:HGNC Symbol;Acc:HGNC:18143] | 8227 | -0.173 | -0.1997 | No |
| 78 | FZD10 | frizzled class receptor 10 [Source:HGNC Symbol;Acc:HGNC:4039] | 8354 | -0.182 | -0.2055 | No |
| 79 | FZD6 | frizzled class receptor 6 [Source:HGNC Symbol;Acc:HGNC:4044] | 8379 | -0.184 | -0.2033 | No |
| 80 | SKP1 | S-phase kinase associated protein 1 [Source:HGNC Symbol;Acc:HGNC:10899] | 8454 | -0.190 | -0.2048 | No |
| 81 | CHP2 | calcineurin like EF-hand protein 2 [Source:HGNC Symbol;Acc:HGNC:24927] | 8573 | -0.199 | -0.2096 | No |
| 82 | PRKCG | protein kinase C gamma [Source:HGNC Symbol;Acc:HGNC:9402] | 8582 | -0.200 | -0.2058 | No |
| 83 | RHOA | ras homolog family member A [Source:HGNC Symbol;Acc:HGNC:667] | 8601 | -0.201 | -0.2027 | No |
| 84 | CER1 | "cerberus 1, DAN family BMP antagonist [Source:HGNC Symbol;Acc:HGNC:1862]" | 8607 | -0.202 | -0.1986 | No |
| 85 | DVL3 | dishevelled segment polarity protein 3 [Source:HGNC Symbol;Acc:HGNC:3087] | 8756 | -0.213 | -0.2055 | No |
| 86 | CAMK2B | calcium/calmodulin dependent protein kinase II beta [Source:HGNC Symbol;Acc:HGNC:1461] | 8782 | -0.215 | -0.2026 | No |
| 87 | WNT16 | Wnt family member 16 [Source:HGNC Symbol;Acc:HGNC:16267] | 8808 | -0.218 | -0.1998 | No |
| 88 | MAPK8 | mitogen-activated protein kinase 8 [Source:HGNC Symbol;Acc:HGNC:6881] | 8956 | -0.228 | -0.2062 | No |
| 89 | SMAD4 | SMAD family member 4 [Source:HGNC Symbol;Acc:HGNC:6770] | 9035 | -0.235 | -0.2071 | No |
| 90 | TBL1Y | transducin beta like 1 Y-linked [Source:HGNC Symbol;Acc:HGNC:18502] | 9416 | -0.266 | -0.2309 | No |
| 91 | PPP2R5B | protein phosphatase 2 regulatory subunit B'beta [Source:HGNC Symbol;Acc:HGNC:9310] | 9554 | -0.279 | -0.2354 | No |
| 92 | FZD3 | frizzled class receptor 3 [Source:HGNC Symbol;Acc:HGNC:4041] | 9560 | -0.279 | -0.2296 | No |
| 93 | MAPK10 | mitogen-activated protein kinase 10 [Source:HGNC Symbol;Acc:HGNC:6872] | 9699 | -0.291 | -0.2339 | No |
| 94 | CUL1 | cullin 1 [Source:HGNC Symbol;Acc:HGNC:2551] | 9762 | -0.297 | -0.2321 | No |
| 95 | CTBP1 | C-terminal binding protein 1 [Source:HGNC Symbol;Acc:HGNC:2494] | 10188 | -0.339 | -0.2579 | No |
| 96 | AXIN1 | axin 1 [Source:HGNC Symbol;Acc:HGNC:903] | 10335 | -0.354 | -0.2614 | Yes |
| 97 | PPP2R5E | protein phosphatase 2 regulatory subunit B'epsilon [Source:HGNC Symbol;Acc:HGNC:9313] | 10392 | -0.360 | -0.2578 | Yes |
| 98 | PPP2R1A | protein phosphatase 2 scaffold subunit Aalpha [Source:HGNC Symbol;Acc:HGNC:9302] | 10395 | -0.360 | -0.2499 | Yes |
| 99 | PORCN | porcupine O-acyltransferase [Source:HGNC Symbol;Acc:HGNC:17652] | 10421 | -0.363 | -0.2438 | Yes |
| 100 | APC | APC regulator of WNT signaling pathway [Source:HGNC Symbol;Acc:HGNC:583] | 10440 | -0.366 | -0.2370 | Yes |
| 101 | PRKACG | protein kinase cAMP-activated catalytic subunit gamma [Source:HGNC Symbol;Acc:HGNC:9382] | 10466 | -0.370 | -0.2307 | Yes |
| 102 | PPP2R1B | protein phosphatase 2 scaffold subunit Abeta [Source:HGNC Symbol;Acc:HGNC:9303] | 10578 | -0.382 | -0.2309 | Yes |
| 103 | PRKACA | protein kinase cAMP-activated catalytic subunit alpha [Source:HGNC Symbol;Acc:HGNC:9380] | 10611 | -0.384 | -0.2249 | Yes |
| 104 | ROCK2 | Rho associated coiled-coil containing protein kinase 2 [Source:HGNC Symbol;Acc:HGNC:10252] | 10648 | -0.388 | -0.2190 | Yes |
| 105 | WNT1 | Wnt family member 1 [Source:HGNC Symbol;Acc:HGNC:12774] | 10706 | -0.394 | -0.2147 | Yes |
| 106 | BTRC | beta-transducin repeat containing E3 ubiquitin protein ligase [Source:HGNC Symbol;Acc:HGNC:1144] | 10836 | -0.409 | -0.2157 | Yes |
| 107 | SOX17 | SRY-box transcription factor 17 [Source:HGNC Symbol;Acc:HGNC:18122] | 10843 | -0.410 | -0.2070 | Yes |
| 108 | CREBBP | CREB binding protein [Source:HGNC Symbol;Acc:HGNC:2348] | 10881 | -0.414 | -0.2007 | Yes |
| 109 | CHP1 | calcineurin like EF-hand protein 1 [Source:HGNC Symbol;Acc:HGNC:17433] | 10940 | -0.423 | -0.1958 | Yes |
| 110 | CXXC4 | CXXC finger protein 4 [Source:HGNC Symbol;Acc:HGNC:24593] | 11061 | -0.440 | -0.1954 | Yes |
| 111 | MMP7 | matrix metallopeptidase 7 [Source:HGNC Symbol;Acc:HGNC:7174] | 11073 | -0.441 | -0.1864 | Yes |
| 112 | GSK3B | glycogen synthase kinase 3 beta [Source:HGNC Symbol;Acc:HGNC:4617] | 11157 | -0.451 | -0.1829 | Yes |
| 113 | FOSL1 | "FOS like 1, AP-1 transcription factor subunit [Source:HGNC Symbol;Acc:HGNC:13718]" | 11222 | -0.460 | -0.1776 | Yes |
| 114 | TBL1X | transducin beta like 1 X-linked [Source:HGNC Symbol;Acc:HGNC:11585] | 11263 | -0.466 | -0.1704 | Yes |
| 115 | PPARD | peroxisome proliferator activated receptor delta [Source:HGNC Symbol;Acc:HGNC:9235] | 11301 | -0.471 | -0.1628 | Yes |
| 116 | CSNK1A1 | casein kinase 1 alpha 1 [Source:HGNC Symbol;Acc:HGNC:2451] | 11310 | -0.472 | -0.1529 | Yes |
| 117 | NFATC4 | nuclear factor of activated T cells 4 [Source:HGNC Symbol;Acc:HGNC:7778] | 11363 | -0.481 | -0.1462 | Yes |
| 118 | CSNK1E | casein kinase 1 epsilon [Source:HGNC Symbol;Acc:HGNC:2453] | 11491 | -0.500 | -0.1450 | Yes |
| 119 | WNT8B | Wnt family member 8B [Source:HGNC Symbol;Acc:HGNC:12789] | 11532 | -0.505 | -0.1369 | Yes |
| 120 | PRKX | protein kinase X-linked [Source:HGNC Symbol;Acc:HGNC:9441] | 11634 | -0.522 | -0.1331 | Yes |
| 121 | EP300 | E1A binding protein p300 [Source:HGNC Symbol;Acc:HGNC:3373] | 11741 | -0.540 | -0.1294 | Yes |
| 122 | CHD8 | chromodomain helicase DNA binding protein 8 [Source:HGNC Symbol;Acc:HGNC:20153] | 11825 | -0.554 | -0.1236 | Yes |
| 123 | CSNK2A1 | casein kinase 2 alpha 1 [Source:HGNC Symbol;Acc:HGNC:2457] | 11895 | -0.570 | -0.1163 | Yes |
| 124 | FBXW11 | F-box and WD repeat domain containing 11 [Source:HGNC Symbol;Acc:HGNC:13607] | 11982 | -0.590 | -0.1098 | Yes |
| 125 | SFRP5 | secreted frizzled related protein 5 [Source:HGNC Symbol;Acc:HGNC:10779] | 11989 | -0.591 | -0.0971 | Yes |
| 126 | ROCK1 | Rho associated coiled-coil containing protein kinase 1 [Source:HGNC Symbol;Acc:HGNC:10251] | 12046 | -0.605 | -0.0880 | Yes |
| 127 | LRP6 | LDL receptor related protein 6 [Source:HGNC Symbol;Acc:HGNC:6698] | 12051 | -0.607 | -0.0748 | Yes |
| 128 | WNT4 | Wnt family member 4 [Source:HGNC Symbol;Acc:HGNC:12783] | 12331 | -0.686 | -0.0814 | Yes |
| 129 | TCF7 | transcription factor 7 [Source:HGNC Symbol;Acc:HGNC:11639] | 12628 | -0.813 | -0.0864 | Yes |
| 130 | PPP3R1 | "protein phosphatase 3 regulatory subunit B, alpha [Source:HGNC Symbol;Acc:HGNC:9317]" | 12653 | -0.829 | -0.0698 | Yes |
| 131 | NFAT5 | nuclear factor of activated T cells 5 [Source:HGNC Symbol;Acc:HGNC:7774] | 12741 | -0.903 | -0.0565 | Yes |
| 132 | MYC | "MYC proto-oncogene, bHLH transcription factor [Source:HGNC Symbol;Acc:HGNC:7553]" | 12801 | -0.970 | -0.0395 | Yes |
| 133 | JUN | "Jun proto-oncogene, AP-1 transcription factor subunit [Source:HGNC Symbol;Acc:HGNC:6204]" | 12813 | -0.994 | -0.0182 | Yes |
| 134 | CTNNB1 | catenin beta 1 [Source:HGNC Symbol;Acc:HGNC:2514] | 12866 | -1.119 | 0.0027 | Yes |
Table: GSEA details [plain text format]

  

Fig 2: KEGG\_WNT\_SIGNALING\_PATHWAY      
 Blue-Pink O' Gram in the Space of the Analyzed GeneSet

  

Fig 3: KEGG\_WNT\_SIGNALING\_PATHWAY: Random ES distribution      
 Gene set null distribution of ES for **KEGG\_WNT\_SIGNALING\_PATHWAY**
